# A Large Lumbar Adnexal Neoplasm Presenting Characteristics of Eccrine Poroma and Poroid Hidradenoma

**DOI:** 10.1155/2017/9865672

**Published:** 2017-09-19

**Authors:** Adamantios Michalinos, Dimitrios Schizas, Antonios Sarakinos, Georgios Athanasiadis, Eleftherios Spartalis, Dimitrios Vlachodimitropoulos, Theodore Troupis

**Affiliations:** ^1^Department of Anatomy, Faculty of Medicine, National and Kapodistrian University of Athens, Athens, Greece; ^2^1st Department of Surgery, Faculty of Medicine, National and Kapodistrian University of Athens, Athens, Greece; ^3^Red Cross Hospital, Athens, Greece; ^4^Laboratory of Experimental Surgery and Surgical Research, University of Athens Medical School, Athens, Greece; ^5^Department of Forensic Medicine and Toxicology, National and Kapodistrian University of Athens, Athens, Greece

## Abstract

Poroma is a rare benign neoplasm that derives from eccrine sweat glands epithelium. Its histological subtypes, with respect to its position within skin layers, are eccrine poroma, hidroacanthoma simplex, poroid hidradenoma, and dermal duct tumor. Poromas commonly exhibit benign clinical behavior as they are usually small and asymptomatic and do not exhibit malignant behavior. Although their histological subtypes share similar histological characteristics, they rarely coexist in the same tumor. In this report we present the case of an unusual poroma in terms of histological and clinical behavior as it was large and presented histological characteristic of both eccrine poroma and poroid hidradenoma. Coexistence of different histologic subtypes in the same tumor indicates simultaneous tumorigenesis, differentiation from one cell type to another, or parallel differentiation from a common progenitor cell. Implications in treatment remain unknown partly due to the rarity of such cases.

## 1. Introduction

Poromas are rare neoplasms, derived from eccrine sweat glands epithelium. They share similar histological and cellular characteristics as monomorphic character of tumor cells, ductal differentiation, and necrosis “en masse.” Poromas are subclassified as eccrine poroma (EP), hidroacanthoma simplex (HS), poroid hidradenoma (PH), and dermal duct tumor (DDT) [1]. When the tumor is confined in epidermis, it is called HS. When it involves basal layer of epidermis and extends into superficial part of the dermis, it is EP. When restricted on dermis and arranged in form of discrete sparse nodules, it is DDT. And finally when it is tumor with solid and cystic components without connection to epidermis, it is called PH [2]. They rarely exhibit malignant behavior [3]. Coexistence of both types in the same tumor is rare and subjected to yet unknown tumorigenesis mechanisms. There are no known differences in clinical image or treatment. This report describes a case of an unusually large neoplasm exhibiting both characteristics of EP and PH.

## 2. Case Presentation

**Figure 1:**
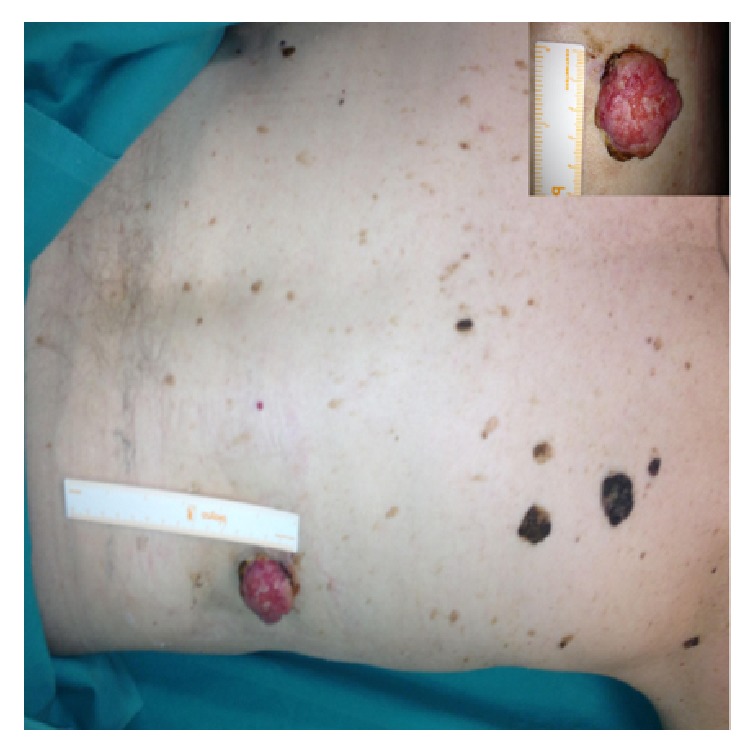
Macroscopic view of the smooth, reddish, and purulent tumor. No signs of infiltration are seen in the surrounding skin.

We present the case of a 79-year-old man with a large (maximum diameter: 4 cm) skin tumor in the right lumbar area. Patient first noted its existence about 8 years ago. The tumor was painless, gradually enlarging, and otherwise asymptomatic. Macroscopically the tumor was pediculated, had about 4 cm maximum diameter, and was attached to the right lumbar area with a 2 cm stem (Figure 1). No deeper abnormalities were found at palpation. Its surface was smooth, reddish, and at some places hemorrhagic. The tumor had a purulent smell but surrounding area did not exhibit any signs of inflammation. The tumor was confronted with local wide excision under regional anesthesia with a lateral margin of 2 cm and a depth margin of 1 cm. No plastic reconstruction was deemed necessary. Patient's course was uneventful. After 3 years of follow-up no recurrence has been noted.

**Figure 2:**
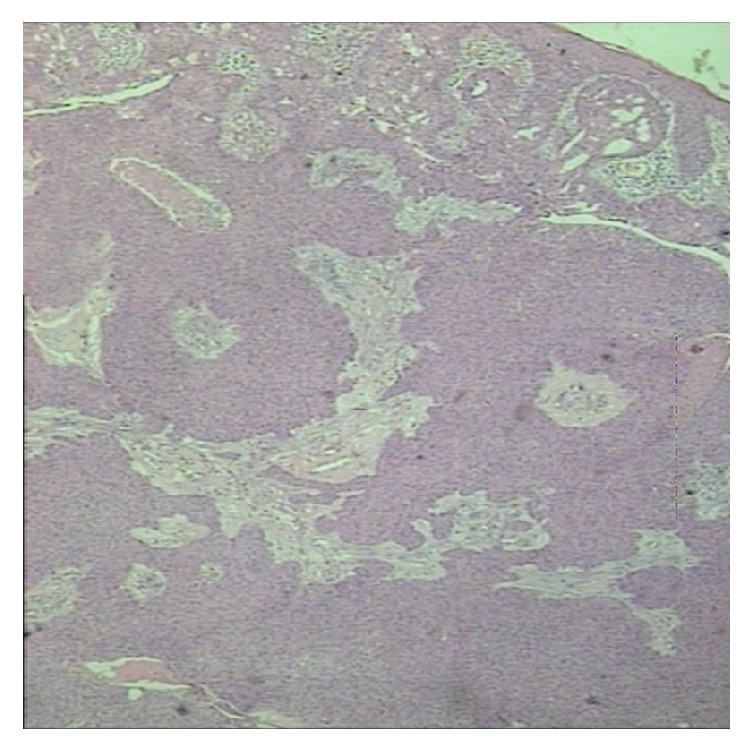
Histological examination of the tumor revealed mixed character of eccrine poroma and poroid hidradenoma (H&E ×25).

**Figure 3:**
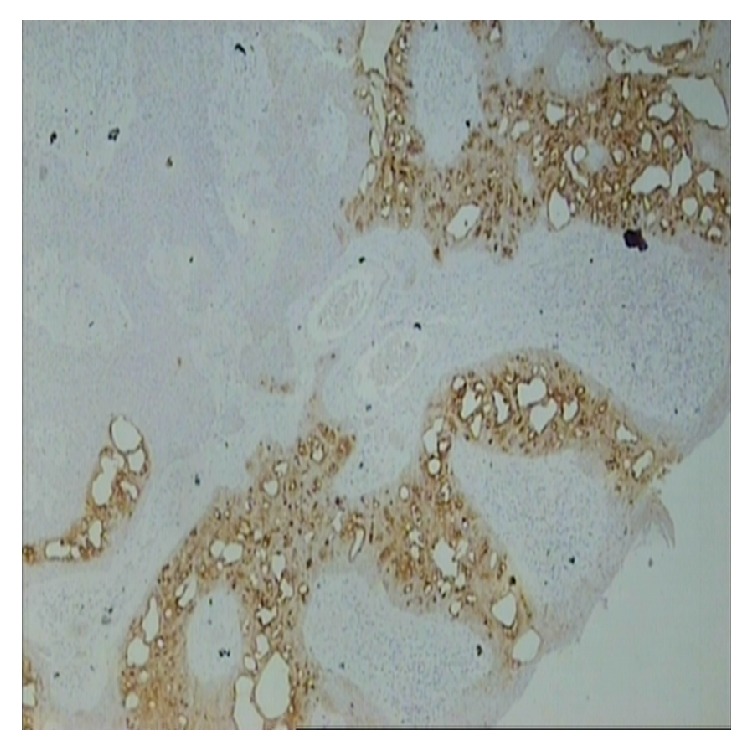
Immunostaining with ck-7 showed focal staining at eccrine poroma sites while areas of poroid hidradenoma were negative (ck-7 ×25).

Histological examination showed a poroma of mixed eccrine poroma and poroid hidradenoma subtypes. Subclassification of poroma types in eccrine poroma and poroid hidradenoma was with respect to their position in dermis layer. Tumor's architecture was lobular with areas of focal necroses and no signs of malignancy (Figure 2). Staining with K7 was focally positive at eccrine poroma sites while Ki-67 was <5% (Figure 3). Excision margins were free.

## 3. Discussion

The term poroma refers to a group of benign adnexal neoplasms that exhibit tubular or distal ductal differentiation. Firstly described by Pinkus et al. [4] in 1956, poromas have been considered as glandular adnexal neoplasms of eccrine lineage. According to Ackerman and Abenoza [1] 4 subtypes exist: the HS, the EP, the DDT, and the PH. These types have identical poroid and cuticular cells and share common histological features such as monomorphism of the nuclei cells, ductal differentiation, and massive necrosis. Poroid cells are thought to derive from luminar cells from upper intradermal duct while cuticular cells are considered reminiscent of normal luminal duct cells [5]. PH differs from EP in having no connection with epidermis. Immunohistochemical staining is similar for eccrine poroma and poroid hidradenoma. PH are found to have significantly fewer CD1a(+) Langerhans cells at tumor islands [3].

Poromas are considered historically as neoplasms of eccrine lineage but later studies suggested that poromas can be of either eccrine or apocrine lineage [6]. These tumors represent approximately 1% of the primary skin lesions. Their malignant counterpart is called porocarcinoma and is much rarer than poroma. Still poromas should be excised as they can undergo malignant differentiation [7]. p53 mutations are thought to play an important role in malignant transformation for poroma to poroid carcinoma [8].

Their etiology is still undefined but inflammation and tissue regeneration might play a role as they are mainly associated with older trauma in the area, scarring, exposure to radiation [9], and immunosuppression [10]. Actinic damage, radiation, trauma, and human papilloma virus have also been implicated at their tumorigenesis [11]. Age of appearance varies but it is more commoner above 40 years of age with an average of 68 years. Commonest location is the head and neck and not the foot as previously believed. Also it is usual in the extremities and the trunk [12]. HS does not occur in palms or soles [5]. Apart from its size, case described showed typical clinical characteristics. Patient referred to no history of trauma or inflammation in the area. Older than usual age can be attributed to longer clinical course.

It is uncommon for two subtypes of poroma to coexist in a single lesion [13]. On the histological examination the lesion described here proved to be adnexal tumor of the skin with features of both eccrine poroma and poroid hidradenoma. A similar lesion has been described by Misago and Kohda [14] and Kakinuma et al. [15] featuring hidroacanthoma simplex, eccrine poroma, and dermal duct tumor features. The coexistence of these tumors is probably attributed to simultaneous tumorigenesis in different parts of the skin and the sweat glands that leads to different expressions of eccrine poroma [2]. This hypothesis is strongly supported by different keratin expression patterns across those tumors [5]. A different explanation could be that tumorigenesis is a time- dependent process. Tumor described in this report existed for at least 8 years and thus it is possible that an initially unique poroid cells population differentiated into two histological subtypes. It is not possible to identify whether it was EP or PH.

## 4. Conclusion

Description of this case provides insight into tumorigenesis cases in these adnexal neoplasms. Coexistence of two different tumor subtypes in a single lesion is rare yet possible and undermines late differences in carcinogenesis paths. Although surgical treatment would not change, clinicians should be prepared for such rare cases and if necessary seek advice on their proper management as further treatment decisions should be taken in an individualized manner. Even more knowledge of tumorigenesis is often provided from such rare and unexpected cases.
